# Applications of radiomics and machine learning for radiotherapy of malignant brain tumors

**DOI:** 10.1007/s00066-020-01626-8

**Published:** 2020-05-11

**Authors:** Martin Kocher, Maximilian I. Ruge, Norbert Galldiks, Philipp Lohmann

**Affiliations:** 1 Institute of Neuroscience and Medicine (INM-3, -4), Research Center Juelich, Wilhelm-Johnen-Straße, 52428 Juelich, Germany; 2 Department of Stereotaxy and Functional Neurosurgery, Center for Neurosurgery, Faculty of Medicine and University Hospital Cologne, Kerpener Str. 62, 50937 Cologne, Germany; 3 Department of Neurology, Faculty of Medicine and University Hospital Cologne, University of Cologne, Kerpener Str. 62, 50937 Cologne, Germany; 4 Center of Integrated Oncology (CIO), Universities of Aachen, Bonn, Cologne and Düsseldorf, Kerpener Str. 62, 50937 Cologne, Germany

**Keywords:** Artificial intelligence, Deep learning, Glioma, Brain metastases, Multiparametric PET/MRI

## Abstract

**Background:**

Magnetic resonance imaging (MRI) and amino acid positron-emission tomography (PET) of the brain contain a vast amount of structural and functional information that can be analyzed by machine learning algorithms and radiomics for the use of radiotherapy in patients with malignant brain tumors.

**Methods:**

This study is based on comprehensive literature research on machine learning and radiomics analyses in neuroimaging and their potential application for radiotherapy in patients with malignant glioma or brain metastases.

**Results:**

Feature-based radiomics and deep learning-based machine learning methods can be used to improve brain tumor diagnostics and automate various steps of radiotherapy planning. In glioma patients, important applications are the determination of WHO grade and molecular markers for integrated diagnosis in patients not eligible for biopsy or resection, automatic image segmentation for target volume planning, prediction of the location of tumor recurrence, and differentiation of pseudoprogression from actual tumor progression. In patients with brain metastases, radiomics is applied for additional detection of smaller brain metastases, accurate segmentation of multiple larger metastases, prediction of local response after radiosurgery, and differentiation of radiation injury from local brain metastasis relapse. Importantly, high diagnostic accuracies of 80–90% can be achieved by most approaches, despite a large variety in terms of applied imaging techniques and computational methods.

**Conclusion:**

Clinical application of automated image analyses based on radiomics and artificial intelligence has a great potential for improving radiotherapy in patients with malignant brain tumors. However, a common problem associated with these techniques is the large variability and the lack of standardization of the methods applied.

## Introduction

Neuroimaging is a field of medical imaging that has attracted the most advanced techniques and methods because of the brain’s complex structure and rich endowment with molecular receptors and targets. Most of the available local treatment options, including radiotherapy and neurosurgery, heavily depend on precise knowledge of tumor type, location, and extent that can only be derived from modern imaging methods such as magnetic resonance imaging (MRI) and positron-emission tomography (PET) [1]. The usual sequence of diagnostic and therapeutic procedures in radiotherapy of malignant brain tumors comprises an initial MRI examination, tissue sampling by biopsy or tumor resection, histopathologic determination of tumor type (e.g., glioma vs. metastasis), tumor grade and expression of molecular markers, segmentation and target volume definition, dose planning, decision on concomitant and adjuvant systemic therapy, and, finally, detection and treatment of local relapses or radiation-induced injury of the brain during follow-up.

Each of these steps can be aided by application of radiomics and image-based machine learning. In many instances, the success of these procedures depends on the complexity level of the applied imaging techniques. Conventional MRI techniques are T1 weighted, T2 weighted, and fluid attenuated inversion recovery (FLAIR) images, while advanced MRI methods include perfusion-weighted imaging (PWI), diffusion-weighted imaging (DWI) including its variants such as diffusion-tensor imaging (DTI), and MR spectroscopy (MRS). The applied machine learning methods mainly comprise feature-based radiomics, where modeling is based on a mathematically predefined set of features extracted from a manually segmented image, and deep learning approaches where the complete model is trained on the imaging data and does not necessarily require a segmented input. However, the applied imaging and computational methods vary substantially and are far from being standardized. The following review aims at providing an overview of the results that were achieved by different machine learning approaches for patients with malignant glioma and metastases.

## Glioma

### Pre-therapeutic classification and molecular characterization

In 2016, the World Health Organization (WHO) published revised guidelines for the classification of tumors of the central nervous system. The most important glioma types in which radiotherapy is integrated into the treatment concept are WHO grade II diffuse astrocytomas and oligodendrogliomas, WHO grade III anaplastic astrocytomas and anaplastic oligodendrogliomas, and WHO grade IV glioblastomas. The classification is based on both tumor histology as well as the presence of a mutation in the isocitrate dehydrogenase (IDH) gene and the loss of heterozygosity (LOH) of the 1p/19q chromosome arms, which allows an integrated diagnosis according to the WHO classification 2016 [2]. Possible treatment strategies for radiotherapy of glioma patients including concomitant and adjuvant chemotherapy are predominantly based on the WHO grade and, more importantly, on molecular characteristics of the tumor [3], namely the IDH genotype [4], the 1p/19q status [5–7], and the O^6^-methylguanine-DNA methyltransferase (MGMT) promoter methylation status [8–10]. The diagnosis of a malignant glioma and the determination of the molecular markers are usually based on tissue samples obtained by tumor resection or stereotactic biopsy. However, approximately 15% of glioma are unresectable [11, 12], and stereotactic biopsy carries a measurable risk for morbidity, especially in the older population [13]. Therefore, several studies have investigated the application of radiomics for determination of WHO grade [14–21] and molecular characteristics [22–40] in glioma. Results of selected reports applying radiomics or other machine learning methods on conventional MRI, advanced MRI, and PET are reported in more detail in the following sections.

#### Determination of WHO grade in patients with newly diagnosed gliomas

Ditmer and colleagues [15] investigated the diagnostic accuracy of histogram-based radiomics analysis of post-contrast T1-weighted MRI for the differentiation of high-grade from low-grade gliomas in 94 patients. The highest diagnostic accuracy was achieved by using the mean of the histograms (area under curve, AUC, 0.9; sensitivity 93%; specificity 86%). However, the dataset was highly unbalanced and parameter combinations were not investigated. Cho and colleagues [14] applied a radiomics approach using different machine learning classifiers for glioma grading based on 285 datasets from the Brain Tumor Segmentation (BraTS) Challenge 2017 [41] comprising pre- and post-contrast T1-weighted, T2-weighted, and FLAIR MRI. After calculation of 468 radiomics features, 5 were selected for use in a random forest classifier that showed the highest AUC of 0.92 after fivefold cross validation.

Besides conventional MRI, several groups have also used advanced MRI methods in combination with radiomics analyses for glioma grading. Sengupta and colleagues [17] used features extracted from conventional MRI and from PWI. The model generated by a support vector machine classifier yielded low classification errors ranging from 3.7% for WHO grade II vs. III up to 9.4% for WHO grade II vs. grade III vs. grade IV. Similarly, Takahashi and colleagues [18] evaluated T2-weighted MRI in combination with DWI for differentiation of glioblastoma from lower-grade glioma in 54 patients. After feature selection, a support vector machine classifier using 6 parameters extracted from DWI demonstrated the best performance in the test dataset (AUC, 0.93). Besides MRI radiomics, Pyka and colleagues evaluated the ability of amino acid PET radiomics using the tracer O‑(2-[^18^F]fluoroethyl)-L-tyrosine (FET) for glioma grading [40]. The combination of textural features calculated from the grey-level neighborhood difference matrix and the metabolic tumor volume yielded a diagnostic accuracy of 85% for the differentiation of WHO grade III and IV gliomas.

Yang and colleagues [21] investigated the usefulness of deep learning-based radiomics using convolutional neural networks (CNNs) for glioma grading in a group of 113 patients. Conventional and advanced MRI including PWI and DWI were available and two commonly used CNNs (AlexNet and GoogLeNet) were explored. The CNNs were applied in two forms. First, only the network structure was taken over and the networks were trained from scratch using the MR images. Second, the networks were used with the connection weights already pretrained on a dataset of 1.2 million non-medical images from “ImageNet,” a large-scale natural image database, and were only finetuned by retraining the first convolutional layer and the final fully connected layers on the MR images. The pretrained, finetuned GoogLeNet showed the best classification accuracy in the test dataset (AUC 0.94). This study demonstrates that pretrained CNNs can be useful for clinical decision-making in patients with gliomas, especially when the number of available patients is low. In summary, assessment of WHO grade in newly diagnosed gliomas by means of machine learning methods achieves an accuracy of approximately 90% and may thus be of clinical benefit in patients unsuitable for resection or biopsy.

#### Determination of IDH genotype and 1p/19q status in patients with newly diagnosed gliomas

Once the diagnosis of a diffuse glioma has been made, usually from tissue sampling, the treatment plan, including dose, type, and fractionation of radiotherapy as well as the sequence of chemotherapy, is mainly determined by the molecular characteristics of the tumor based on the WHO classification [3]. Many groups have shown that these may also be derived non-invasively from imaging by application of machine learning [22–25, 33–35, 38, 39, 42, 43]. Zhang and co-workers [43] used conventional MRI as well as DWI and extracted a total of 2970 features from 120 patients with WHO grade III and IV gliomas. Finally, a subset of 386 features was used to build a model based on a random forest classification, resulting in an accuracy of 89% for IDH prediction in the validation dataset. In WHO grade II and III gliomas, Zhou and colleagues [39] showed that a logistic regression model with only three features obtained from conventional MRI of 165 patients predicted the IDH genotype as well as the 1p/19q co-deletion status with AUC values of 0.86 and 0.96, respectively. In a subsequent study [38], Zhou and colleagues extracted radiomics features from conventional MRI of a large multicentric dataset of more than 500 patients. The model was built using a random forest classifier and validated using another set of MR images from The Cancer Imaging Archive (TCIA). The IDH genotype could be predicted with an AUC of 0.92 in the test cohort. Lu and colleagues [34] used conventional MRI data from 214 glioma patients from TCIA. Additionally, 70 patients with MRI scans from different institutions were used for model testing. A multi-level machine learning model based on support vector machine classifiers was used and allowed classification of IDH and 1p/19q status with accuracies of 88% and 96%, respectively. Lohmann and colleagues [33] used FET PET radiomics for preoperative prediction of IDH genotype in a cohort of 84 glioma patients and identified a simple two-parameter logistic regression model that achieved a diagnostic accuracy of 80% after 10-fold cross validation. A subgroup analysis of 28 patients measured on a high-resolution BrainPET scanner showed the highest accuracy of 86% after 10-fold cross validation.

Again, deep learning-based radiomics approaches have also been investigated for prediction of molecular characteristics in gliomas. Chang and co-workers [22] used a residual CNN for IDH genotype prediction on conventional MRI from a large cohort of 496 glioma patients from three different institutions and achieved an accuracy of 86% in an independent test set. The accuracy could be further increased to 89% by the incorporation of age at diagnosis. Eichinger and colleagues [24] used a set of local binary pattern features extracted from T2-weighted MRI and DTI for training of a CNN with a single hidden layer. The prediction of IDH genotype yielded a diagnostic accuracy of 95% in the test dataset. These results suggest that radiomics has the potential to substitute neuropathological assessment of IDH mutation and 1p/19q LOH status in glioma patients in whom tissue sampling is not feasible.

#### Determination of MGMT promoter methylation status

The methylation status of the MGMT promoter is of great value for predicting the response to alkylating chemotherapy and has also been determined using advanced image analyses. Korfiatis and colleagues [28] predicted the MGMT promoter methylation status in patients with glioblastoma with a sensitivity of 80% and a specificity of 81% using a four-parameter model based on T2-weighted MRI. Xi and co-workers [36] calculated 1665 radiomics features from conventional MRI and generated a model from a subset of 36 features, resulting in an accuracy of 87% in the validation data and 80% in a test dataset. Similarly, Li and colleagues [31] extracted 1705 radiomics features from conventional MRI and built a predictive random forest model using a subset of six features. The final model resulted in an AUC of 0.88. The combination of clinical factors with radiomic features did not further improve model performance. Kong and colleagues [27] used FDG PET-based radiomics in 107 patients with primary glioma and extracted a total of 1561 features for prediction of MGMT promoter methylation status. Five radiomics features were finally selected to construct the radiomics signature using a support vector machine classifier. The model achieved an AUC of 0.94 in the validation and 0.86 in the test cohort.

By means of a bidirectional, recurrent CNN that leverages the spatial aspects of three-dimensional MRI scans, Han and Kamdar [25] obtained an accuracy of 67% in the validation dataset and 62% in the test data. Korfiatis and colleagues [29] compared three different residual deep neural network (ResNet) architectures for the prediction of MGMT promoter methylation status based on conventional MRI data from 155 patients. The authors found that the ResNet50 with a 50-layer architecture was the best-performing model, achieving an accuracy of 95% in the test dataset. Thus, promising results also exist regarding non-invasive, image-based assessment of MGMT promoter methylation status.

### Image segmentation and delineation of planning target volumes

Malignant gliomas tend to grow in typical patterns and induce a number of characteristic tissue changes. The main tumor compartments, also called “segments” in image processing, comprise the necrotic core, contrast-enhancing tumor, non-enhancing tumor, and perifocal edema. Fast and reliable labeling (contouring) of these segments is a crucial task in many areas of neuro-oncology such as radiotherapy and image-based follow-up [44]. Numerous algorithms have been developed for this purpose [45] and a periodically held challenge (the Multimodal Brain Tumor Image Segmentation Benchmark, BRATS) has been set up to compare their efficacy [41, 46]. Today, the best-performing tools are usually based on CNNs [47, 48], which achieve high segmentation accuracies (Dice similarity coefficients) where almost 90% of the voxels are correctly labeled, which is the order of magnitude that experienced physicians can achieve [44]. In radiotherapy planning for malignant glioma, delineation of gross tumor volume (GTV) and clinical target volume (CTV) could in principle be automatically performed by these software tools [49]. A basic requirement is that segmentation should not only work for untreated tumors but also in less well-defined situations, e.g., after tumor resection, which is the most common situation in radiotherapy for malignant glioma. Vendors of radiotherapy planning systems should aim at integration of these tools into their software in order to accelerate and standardize the contouring process. A powerful segmentation toolkit for scientific use is publicly available at: https://github.com/neuronflow/BraTS-Toolkit.

### Prediction of local relapse location after radiotherapy

In malignant glioma, approximately 80–90% of recurrences that develop after macroscopic tumor resection and postoperative local irradiation with doses of approximately 60 Gy are located within a distance of 2 cm to the margin of the resection cavity and will occur within 6–9 months after initiation of radiotherapy [50–54]. This observation was used for recommending the size of the geometrical margin between the CTV and the GTV in both European and North American guidelines [55, 56]. However, this procedure still results in considerably large volumes of irradiated brain, and almost all attempts to escalate the radiation dose up to 80–90 Gy within a geometrically defined CTV have failed to improve the prognosis of these patients [57–60]. Assuming that the direction of tumor spread is foreseeable and that a dose–response relationship exists for malignant glioma, prediction of the precise location of a recurrence would be of significant value. Ideally, a so-called tumor infiltration map of the peritumoral region should be generated that depicts the areas with the highest risk of recurrence, which, in turn, could be targeted by higher doses. Several approaches including conventional imaging analysis methods and radiomics have been applied to achieve this goal.

Amino acid PET was used for definition of the CTV in a small study with focal dose escalation to 72 Gy. Although the results were encouraging in terms of survival [61], there was only a small overlap between the PET signals at recurrence and the PET signals used for dose planning [62]. In a comparable study, 63% of the recurrent tumor volume was located outside the PET-defined GTV [63]. However, it was shown that the GTV–CTV margins could be reduced by 4 mm for the PET-defined GTV in comparison to the MRI-defined GTV in order to include 100% of the recurrences.

In addition to PET, advanced MR imaging techniques have been applied for predicting the spread of glioma cells. By use of DWI and DTI, regions with restricted diffusion due to increased cellular density can be identified. Indeed, fractional anisotropy (FA), a measure of directed diffusion, was found to be significantly lower in regions with later tumor recurrence [64]. Also, areas of the preoperative peritumoral region with a lowered apparent diffusion coefficient (ADC) overlapped with the region of the later recurrences by 60% [65]. DTI and further advanced MR can also be used to depict the orientation and density of white-matter tracts in the vicinity of the macroscopic tumor. By this approach, it was shown that tumor growth follows the tracts to some extent [66] and that this fact can be used to foresee which brain regions are predominantly affected by recurrent tumor growth [67].

However, the most promising approaches are probably those that use advanced, multiparametric MR imaging in conjunction with radiomics and machine learning. In a series of investigations, a group from the University of Philadelphia applied conventional and advanced MR techniques (pre- and post-contrast T1-weighted MRI, T2-weighted MRI, PWI, and DTI) to determine FA, radial diffusivity, axial diffusivity, ADC, relative cerebral blood volume, and a number of associated first-and second-order features in each voxel of the peritumoral region. A model was trained to predict the voxel’s risk for being involved in a recurrent tumor, which resulted in a tumor infiltration map that had an overall accuracy of approximately 90% [68–70]. If these maps were routinely available, postoperative radiotherapy of malignant glioma could be far more individualized compared to the present practice.

### Prediction of progression-free and overall survival

In radiotherapy planning, not only the putative location of a future recurrence, but also the time interval to progression is of major importance. Methods including radiomics and machine learning have therefore also been used to predict progression-free (PFS) and overall survival (OS), which are both closely related to the time to progression. When using radiomic features from standard preoperative MRI of the primary tumor or the postoperative peritumoral region, predictive models for PFS and OS were developed that outperformed those based on clinical features alone [71, 72]. Also, the performance of these models improved significantly when using advanced MR imaging features, e.g., from PWI or DTI, and combining them with clinical factors [73–76]. In the era of prognostic and predictive molecular markers, the imaging features may seem of less importance. However, Kickingereder and colleagues demonstrated in a cohort of IDH-wildtype glioblastoma patients with known MGMT methylation status that the addition of radiomic features to a comprehensive model comprising clinical, therapeutic, and molecular features could still improve the prediction accuracy [74].

### Differentiation of tumor progression from pseudoprogression

The term pseudoprogression describes the occurrence of a progressive enhancing lesion on MRI within 12 weeks after radiotherapy alone or radiotherapy with concomitant and adjuvant temozolomide chemotherapy in patients with malignant gliomas with spontaneous improvement without any treatment change [77, 78]. Due to its clinical importance, this time-dependent definition of pseudoprogression was included in the recommendations of the Response Assessment in Neuro-Oncology (RANO) working group [79]. Several studies have already demonstrated the usefulness of amino acid PET as well as PWI for this challenging clinical task [77, 80–82]. However, radiomics and machine learning might add important diagnostic information to further improve the diagnostic performance.

Hu and colleagues [83] used conventional and advanced MRI including DWI and PWI for differentiation of tumor progression from pseudoprogression in 31 patients who underwent radiochemotherapy after surgical resection. An eight-dimensional feature vector was constructed, and a support vector machine classifier yielded an AUC of 0.94 (sensitivity 90%; specificity 94%). These findings were confirmed by the study from Kim and colleagues [84]. Therein, a multiparametric model incorporating conventional MRI in combination with DWI and PWI showed a significantly better performance than other models based on single imaging contrasts with an AUC of 0.85 in the test dataset.

FET PET radiomics has also been investigated for differentiation of tumor progression from pseudoprogression. Kebir and co-workers [85] used FET PET scans of 14 patients and applied an unsupervised consensus clustering algorithm for the diagnosis of pseudoprogression, resulting in a diagnostic accuracy of 75%. However, the number of patients was very low and the model was not further validated. Lohmann and colleagues [86] also investigated the potential of FET PET radiomics for this purpose. Thirty-five glioblastoma patients with imaging findings suspicious for pseudoprogression within 12 weeks after completion of radiochemotherapy were included. The final logistic regression model used three textural features and yielded a diagnostic accuracy of 92% after 10-fold cross validation in the training and 86% in the test dataset. Again, the model has to prove its performance in a larger cohort study.

A few studies have applied deep learning-based radiomics to classify seemingly progressing lesions. Jang and co-workers [87] used post-contrast MR images from 78 patients from two institutions. The developed CNN incorporated both clinical and imaging features and achieved an AUC of 0.83 in the test dataset. Interestingly, models based solely on clinical or imaging features showed inferior performance, highlighting the importance of incorporation of clinical features. Bachhi and colleagues [88] used a CNN based on conventional MRI and DWI. In a cohort of 55 patients, the CNN model based on DWI and FLAIR sequences in combination achieved the highest diagnostic accuracy of 82% in the test dataset. Akbari and colleagues [89] also used multiparametric MRI data consisting of conventional MRI as well as DWI and PWI in a cohort of 63 patients where detailed histopathological confirmation of the diagnosis was available for all patients. Classical radiomics features as well as deep learning-based features were combined for classification using support vector machines. The final model achieved a diagnostic accuracy for the diagnosis of pseudoprogression of 87% (AUC 0.92) after leave-one-out cross validation. The accuracy in the inter-institutional test cohort was 75% (AUC 0.8). Of note, the workflow can be easily adapted, as the authors used a freely available software toolkit (Cancer Imaging Phenomics Toolkit). Recently, Li and co-workers [90] introduced a novel feature-learning method based on deep convolutional generative adversarial networks (DCGAN) and a CNN (AlexNet), termed DC-AL GAN. Discriminative features identified by DC-AL GAN were used for classification by support vector machines, resulting in a diagnostic accuracy of 92% (AUC, 0.95) after 10-fold cross validation.

### Differential diagnosis

Glioblastoma and brain metastases are the two most common malignant brain tumors in adults and often present similar clinical and imaging characteristics on conventional MRI [91, 92]. Consequently, differential diagnosis based solely on clinical presentation and MRI alone is often challenging.

Qian and colleagues [93] addressed this question using MRI radiomics. A large group of 412 patients with untreated brain metastases and treatment-naive, newly diagnosed glioblastoma was divided into a training and a test cohort. Tumors were segmented manually and 1303 radiomic features were calculated on contrast-enhanced MR images. The best classifier that showed a high predictive performance in the test cohort (AUC 0.90) was a support vector machine algorithm that used the least absolute shrinkage and selection operator (LASSO) for feature selection. Also, the classifier showed a better performance than experienced neuroradiologists.

Artzi and colleagues [94] extracted 760 radiomics features from contrast-enhanced MR images of 439 patients with brain metastases or glioblastoma. After image preprocessing and semi-automatic tumor segmentation using a region-growing algorithm, feature selection and model generation was performed. Interestingly, the authors identified the same support vector machine algorithm as the study by Qian and colleagues described above to have the highest predictive performance in the test cohort (AUC 0.96) for the differentiation of brain metastases from glioblastoma.

## Brain metastases

### Detection and automatic segmentation of brain metastases

Nowadays, the dominant type of radiotherapy for a limited number and limited total volume of brain metastases is radiosurgery [95]. Particularly when multiple metastases are present, manual contouring is a laborious task. Automatic segmentation of brain metastases for use in radiosurgery planning differs in several aspects from that of gliomas. As metastases are thought to have a low infiltrative potential, definition of the GTV is mainly based on the T1-weighted, contrast-enhanced MR images with no or only small GTV–CTV margins, while other tumor segments are usually not considered. However, some metastases leave the blood–brain barrier intact, while others grow adjacent to other contrast-enhancing structures or resemble small vessels in appearance. Therefore, automatic segmentation algorithms should be able to both reliably identify brain metastases (measured by the detection rate and the false-positive rate) and to accurately contour them (measured by the Dice similarity coefficient). To achieve this goal, most groups adapted existing CNN solutions. Liu and colleagues applied a modified version of the widely used DeepMedic CNN for use with small metastases (<1.5 cm) and achieved an average Dice coefficient of 0.67 on contrast-enhanced MR images [96]. The same CNN was used in a dataset were contrast-enhanced and FLAIR MR images were available and achieved a Dice coefficient of 0.79 with a typical detection rate of 93%, which came at the cost of a false-positive rate of 7.8 metastases per patient [97]. A group from Stanford used a CNN based on GoogLeNet on contrast-enhanced and FLAIR MR images and achieved a Dice coefficient of 0.79. However, the sensitivity was only 50% for metastases <7 mm but 100% for lesions >22 mm at an average false-positive rate of 8.3 metastases per patient. In summary, the contouring performance of these automated systems seems to be at the edge of suitability for clinical use, while secure detection of small metastases is at present only possible if a considerable number of falsely labeled non-tumor regions is accepted.

### Prediction of local response after radiosurgery of brain metastases

It is widely believed that the main determinants of local response to radiosurgery are tumor volume and prescription dose; however, the response to radiosurgery is still difficult to predict in a subset of brain metastases. This may be partly due to the more radioresistant tumor cells residing at the border of a necrotic core that is present in a proportion of the metastases, and to differences in the tumor vasculature that takes part in promoting the response to single high-dose irradiation [98]. As these tumor properties may be reflected in the radiological appearance of the metastasis and its surrounding area, radiomics is a promising tool for predicting response after radiosurgery. Indeed, simple features such as the presence of a necrotic core [99], the fraction of contrast-enhancing tumor tissue [100], or the extension of the perifocal edema [101] have been shown to impact on response or survival. The experience with more advanced imaging methods and radiomics is limited so far. However, by training a simple CNN on image patches from a cranial computed tomography, Cha and co-workers were able to classify tumors as responding vs. non-responding with an AUC of approximately 0.8 [102]. In a recent report, a classic radiomics approach was applied with features extracted from contrast-enhanced and FLAIR MR images of the core metastasis and its immediate vicinity. By adding any of the top-ranked radiomic features to a model based on pure clinical and dosimetric factors (dose, isodose, diameter, number and location of metastases, previous whole-brain radiotherapy), the AUC for predicting response could be increased from approximately 0.7 to 0.8 [103].

### Differentiation of radiation injury from local brain metastasis relapse

Following radiosurgery for brain metastases, radiation injury of the brain tissue may develop in approximately 5–20% of patients [104]. Radiation injury is usually suspected if new contrast-enhancing lesions appear in or adjacent to the GTV and is often indistinguishable from local brain metastasis relapse using conventional MRI alone. The pathological mechanisms involved in these two processes differ substantially [105] and should therefore be distinguishable by a radiomics analysis of the associated image changes.

Peng and colleagues [106] evaluated the usefulness of MRI radiomics for this important question. Sixty-six patients with 82 lesions treated with stereotactic radiosurgery and imaging findings on contrast-enhanced and FLAIR MRI suspicious for tumor recurrence were included in the study. Fifty-one radiomics features (3 shape features, 14 histogram-based features, and 34 textural features) were extracted for each lesion on each MRI contrast. Models were generated using the IsoSVM algorithm, which performs both feature selection and classification [107]. No separate dataset was available for model testing. However, cross validation was performed to assess overall model performance. The model reached an area under the receiver operating characteristic curve (AUC) of 0.81, with a specificity of 65% and a sensitivity of 87%. On the contrary, experienced radiologists could only classify 73% of the cases, with a sensitivity of 97% and a specificity of only 19%.

Similarly, Zhang and colleagues [108] used pre- and post-contrast T1-weighted MR images, T2, and FLAIR from 87 patients after Gamma Knife (Elekta, Stockholm, Sweden) radiosurgery to calculate 285 radiomics features. Interestingly, imaging data from two timepoints were available, so that the authors also investigated feature reproducibility to identify a feature subset with reproducible values. Changes in radiomics features (so-called delta radiomics) from one follow-up timepoint to the next were evaluated and used for differentiation of radiation necrosis and tumor progression. The final model generated by an ensemble classifier had an overall predictive accuracy of 73% and an AUC of 0.73 after cross validation. Again, no separate test dataset was available.

Besides MRI, amino acid PET has also been investigated to evaluate radiomics for the differentiation of treatment-related changes from brain metastasis recurrence. It has been demonstrated that evaluation of the time–activity curves (TAC) that represent the tracer uptake over time is helpful for differentiation of treatment-related changes from brain metastasis recurrence [109]. However, this requires a time-consuming dynamic FET PET scan of at least 40 min acquisition time or more. Therefore, Lohmann and colleagues [110] calculated 62 textural parameters on static FET PET scans from 47 patients with MRI findings suspicious for tumor recurrence after radiosurgery. Combinations of conventional FET PET and textural features were investigated using ROC analysis without prior feature selection. The diagnostic accuracy of conventional FET PET parameters was in the range of 81–83% and could be slightly increased to 85% when combined with textural features. However, no dataset for validation or testing was available. In a subsequent study, Lohmann and colleagues [111] investigated the value of combining FET PET and MRI radiomics for the differentiation of treatment-related changes from brain metastasis recurrence. Fifty-two patients with newly or progressive contrast-enhancing lesions on MRI after radiotherapy were additionally investigated using FET PET. After feature selection, logistic regression models limited to a maximum of five parameters to avoid over-fitting were generated for the combined PET/MRI features and for each modality separately and validated using cross validation; no test dataset was available. The highest diagnostic accuracy of 89% (specificity 96%, sensitivity 85%) was achieved by the combination of MRI and FET PET features, suggesting that the combined FET PET/MRI radiomics analysis encoded more diagnostic information than either modality alone. In summary, both MRI- and PET-based radiomics achieve classification accuracies far above chance for differentiation of radiation injury from tumor recurrence and thus demonstrate the potential of this approach.

## Conclusion

Feature-based radiomics and deep learning-based machine learning methods can be used to facilitate and automate many of the diagnostic and therapeutic steps needed in radiotherapy of malignant brain tumors with high accuracy. A common problem associated with the application of the derived models is the large variability and the lack of standardization of both image acquisition and computational methods [112]. From the perspective of radiotherapy, the most promising applications include automatic segmentation of the target volumes, prediction of the location and timepoint of local recurrences of glioma by tumor infiltration maps, and differentiation of treatment-related tissue changes from true recurrences.
